# Disease-causing mutations in the XIAP BIR2 domain impair NOD2-dependent immune signalling

**DOI:** 10.1002/emmm.201303090

**Published:** 2013-07-01

**Authors:** Rune Busk Damgaard, Berthe Katrine Fiil, Carsten Speckmann, Monica Yabal, Udo zur Stadt, Simon Bekker-Jensen, Philipp J Jost, Stephan Ehl, Niels Mailand, Mads Gyrd-Hansen

**Affiliations:** 1 Department of Disease BiologyNovo Nordisk Foundation Center for Protein Research, University of CopenhagenCopenhagen, Denmark; 2 CCI – Centre of Chronic Immunodeficiency, University Hospital FreiburgFreiburg, Germany; 3 III. Medizinische KlinikKlinikum rechts der Isar, Technische Universität MünchenMunich, Germany; 4 Center for Diagnostic, University Medical Center Hamburg EppendorfHamburg, Germany

**Keywords:** BIR2, NOD2, Smac mimetic compounds, XIAP, XLP2

## Abstract

X-linked Inhibitor of Apoptosis (XIAP) is an essential ubiquitin ligase for pro-inflammatory signalling downstream of the nucleotide-binding oligomerization domain containing (NOD)-1 and -2 pattern recognition receptors. Mutations in *XIAP* cause X-linked lymphoproliferative syndrome type-2 (XLP2), an immunodeficiency associated with a potentially fatal deregulation of the immune system, whose aetiology is not well understood. Here, we identify the XIAP baculovirus IAP repeat (BIR)2 domain as a hotspot for missense mutations in XLP2. We demonstrate that XLP2-BIR2 mutations severely impair NOD1/2-dependent immune signalling in primary cells from XLP2 patients and in reconstituted XIAP-deficient cell lines. XLP2-BIR2 mutations abolish the XIAP-RIPK2 interaction resulting in impaired ubiquitylation of RIPK2 and recruitment of linear ubiquitin chain assembly complex (LUBAC) to the NOD2-complex. We show that the RIPK2 binding site in XIAP overlaps with the BIR2 IBM-binding pocket and find that a bivalent Smac mimetic compound (SMC) potently antagonises XIAP function downstream of NOD2 to limit signalling. These findings suggest that impaired immune signalling in response to NOD1/2 stimulation is a general defect in XLP2 and demonstrate that the XIAP BIR2-RIPK2 interaction may be targeted pharmacologically to modulate inflammatory signalling.

The X-linked lymphoproliferative syndrome type-2 is an immunodeficiency disease caused by mutations in the XIAP gene. BIR2 domain mutations in patients impair RIPK2 binding and NOD2-dependent innate immune signaling, explaining some of the pathology.

## INTRODUCTION

Disease-causing mutations in *XIAP/BIRC4* were first described in 2006 in families with patients suffering from X-linked lymphoproliferative syndrome (XLP) with no mutations in the *SH2D1A* gene encoding SAP (Rigaud et al, 2006). Classical XLP due to SAP deficiency (XLP1) is characterized by susceptibility to fulminant Epstein-Barr virus (EBV) infection, frequently leading to haemophagocytic lymphohistiocytosis (HLH), development of lymphoma and hypogammaglobulinemia (Purtilo et al, 1975). XLP2 caused by mutation in *XIAP* shares the susceptibility to EBV with a high risk of HLH, but no patient with lymphoma has so far been reported (Filipovich et al, 2010; Pachlopnik Schmid et al, 2011; Yang et al, 2012). Moreover, severe chronic colitis, hepatitis or persistent splenomegaly are increasingly reported as initial and even as the only clinical manifestations of patients with *XIAP* mutations [(Worthey et al, 2011), Carsten Speckmann et al, in preparation]. The molecular basis of these inflammatory manifestations remains poorly characterized.

The best described cellular function of XIAP is its role in limiting apoptosis through inhibition of apoptotic caspases (Gyrd-Hansen & Meier, 2010) and, as recently reported by us and others, its role in facilitating innate immune signalling downstream of the NOD1 and NOD2 bacterial sensors (Bauler et al, 2008; Damgaard et al, 2012; Krieg et al, 2009; Lipinski et al, 2012). Caspase regulation is mediated by the N-terminal part of XIAP composed of three baculovirus IAP repeat (BIR) domains. BIR domains mediate interactions with proteins that contain an IAP binding motif (IBM) as well as other non-IBM type protein interactions (Gyrd-Hansen & Meier, 2010). IBMs are four-amino acid motifs starting with an N-terminal alanine and are present in several proteins including the processed, mature form of the mitochondrial factor Second mitochondria-derived activator of caspases (Smac; also known as direct IAP binding protein with low pI) and in cleavage-activated caspases. The XIAP BIR2 binds to the IBM in active caspase-3 and -7, and this aids the inhibition of the caspases through the linker region immediately N-terminal to the BIR2 domain (Scott et al, 2005).

XIAP's role in NOD1/2 signalling relies on its ubiquitin (Ub) ligase activity provided by the C-terminal RING domain (Damgaard et al, 2012). NOD2 is a member of the NOD-like receptor family, which also includes NOD1 and NLRPs, and is particularly important for immune regulation at mucosal surfaces (Casanova & Abel, 2009; Chen et al, 2009). Accordingly, *NOD2* was the first identified susceptibility gene for the inflammatory bowel disease termed Crohn's disease (Van Limbergen et al, 2009). Activation of NOD2 by the peptidoglycan component muramyl dipeptide (MDP) in the bacterial cell wall leads to recruitment of RIPK2 and the Ub ligases XIAP, cIAP1 and cIAP2 (Bertrand et al, 2009; Damgaard et al, 2012). This triggers Ub-dependent signalling events that activate mitogen-activated protein (MAP) kinases and the NF-κB-activating IκB kinase (IKK) complex composed of IKKα, IKKβ and NEMO (also termed IKKγ) (Beug et al, 2012; Damgaard & Gyrd-Hansen, 2011). XIAP conjugates Ub chains on RIPK2 together with cIAP1/2 to recruit and enable the activation of the TAK1-TAB1/2/3 and IKK kinase complexes. Full activation of the IKK complex, additionally, requires the presence of Ub chains linked via methionine 1 (M1-linked; also termed linear Ub chains) that are conjugated by the linear ubiquitin chain assembly complex (LUBAC) (Haas et al, 2009; Rahighi et al, 2009; Tokunaga et al, 2009). In turn, IKK phosphorylates IκBα to enable nuclear translocation of NF-κB transcription factors, transcription of NF-κB target genes and production of pro-inflammatory cytokines and chemokines (Bonizzi & Karin, 2004). LUBAC is a trimeric complex composed of the catalytic subunit HOIP and two adaptors HOIL-1 and SHARPIN (Gerlach et al, 2011; Ikeda et al, 2011; Tokunaga et al, 2011), and it is recruited to the NOD2 signalling complex by Ub chains conjugated by XIAP (Damgaard et al, 2012).

Smac mimetic compounds (SMCs) are potent antagonists of IAP proteins and sensitize cancer cells to cell death induced by cytotoxic compounds and by TNF-receptor super family receptor ligands, including TNF. Recently, SMCs have additionally been demonstrated to deregulate inflammatory signalling downstream of TNF-receptor 1 (TNF-R1) and toll-like receptors, and to cause inappropriate activation of the NLRP1/3-inflammasome (Bertrand et al, 2008; Tseng et al, 2010; Vince et al, 2012; Wang et al, 2008). SMCs are designed to bind type-III BIR domains (BIR3 domain in cIAP1/cIAP2/XIAP), and they induce rapid auto-ubiquitylation and proteasomal degradation of cIAP1/2 by activating their Ub ligase activity (Dueber et al, 2011; Feltham et al, 2011; Gaither et al, 2007; Petersen et al, 2007; Varfolomeev et al, 2007; Vince et al, 2007). Contrary to this, SMCs do not activate XIAP's Ub ligase activity or cause its proteasomal degradation (Nakatani et al, 2013; Varfolomeev et al, 2007; Vince et al, 2007). How SMCs may affect XIAP's function in cellular signalling is currently not well understood although an SMC recently was reported to interfere with RIPK2 binding *in vitro* (Krieg et al, 2009).

Here, we provide evidence that XLP2-causing mutations in the XIAP BIR2 domain, similar to RING domain mutations, impair NOD1/2-dependent immune signalling. We show that XLP2-BIR2 mutations abolish RIPK2 binding and that this impairs XIAP-mediated ubiquitylation of RIPK2 and NOD2-dependent induction of NF-κB target genes. Consistently, the SMC Compound A antagonized RIPK2 binding, RIPK2 ubiquitylation and NOD1/2-dependent activation of NF-κB. We conclude that defective NOD1/2 signalling is a common immune defect in XLP2 and thus may contribute to the pathogenesis, and propose that certain SMCs may be used to modulate NOD2-mediated inflammation.

## RESULTS

### XLP2-derived BIR2 mutations abrogate NOD2-dependent signalling

**Figure 1:**
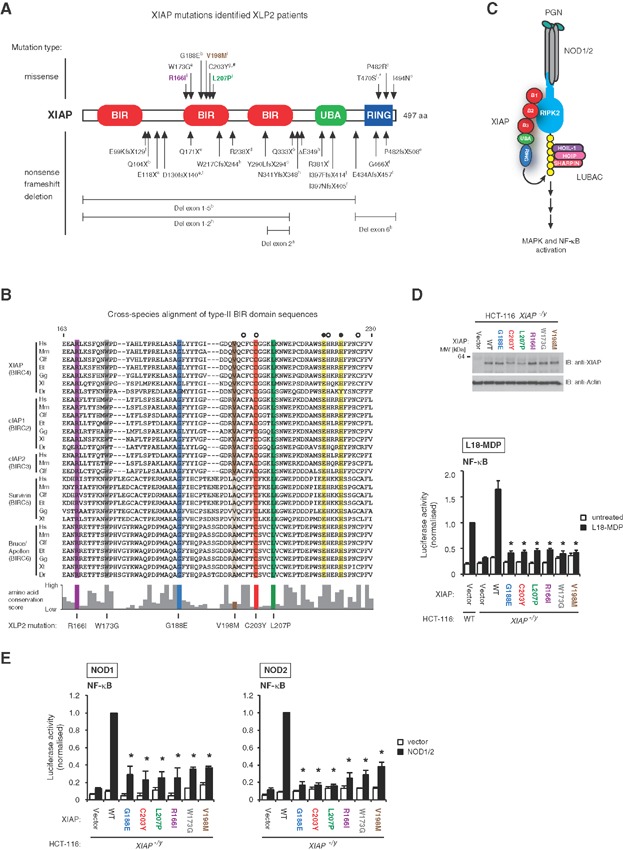
**XLP2-causing BIR2 mutations abrogate NOD1/2 signalling**Schematic showing position and type of XIAP mutations identified in XLP2 patients. Superscript letters refer to the original report of the mutation: a, (Rigaud et al, 2006); b, (Marsh et al, 2009); c, (Marsh et al, 2010); d, (Zhao et al, 2010); e, (Filipovich et al, 2010); f, (Pachlopnik Schmid et al, 2011); g, (Worthey et al, 2011); h, (Yang et al, 2012). (*) Denotes that the mutation is listed as an SNP. Number sign (#) indicates that the mutation was incorrectly annotated in the original report.Amino acid sequences of type-II BIR domains of IAPs were aligned using ClustalX. Graph below aligned sequences shows conservation of amino acid residues. Filled circles denote the E219 and H223 (XIAP numbering) residues that define type-II BIR domains. Open circles indicate residues involved in coordinating the Zn^2+^ ion. XLP2-BIR2 mutations studied here are indicated in colour.Schematic view of XIAP's role in NOD1/2 signalling. In response to NOD1/2 activation by peptidoglycans (PGN), XIAP is recruited to the signalling complex by RIPK2 where it ubiquitylates RIPK2. This enhances recruitment of LUBAC and facilitates activation of NF-κB.NF-κB activity in lysates of WT and reconstituted XIAP-deficient HCT-116 cells. Cells were transfected as indicated and stimulated with L18-MDP (200 ng/mL) for 24 h. Data represent mean + s.e.m. (*n* = 4–6). (*) Indicates *P* = 0.0001 for G188E, *P* = 0.0001 for C203Y, *P* = 0.0001 for L207P, *P* = 0.0002 for R166I, *P* = 0.0009 for W173G, *P* = 0.0001 for V198M, all vs. WT. Expression of XIAP variants in XIAP-deficient HCT-116 cells were analysed by immunoblotting.NF-κB activity in lysates of WT and XIAP-deficient HCT-116 cells co-transfected with XIAP and NOD1 or NOD2 plasmids. Data represent mean + s.e.m. (*n* = 3–4). In NOD1 transfections (*) indicates *P* = 0.006 for G188E, *P* = 0.006 for C203Y, *P* = 0.0001 for L207P, *P* = 0.008 for R166I, *P* = 8.9E-05 for W173G, *P* = 2.9E-05 for V198M, all vs. WT. In NOD2 transfections (*) indicates *P* = 0.0002 for G188E, *P* = 4.6E-05 for C203Y, *P* = 4.5E-05 for L207P, *P* = 0.008 for R166I, *P* = 0.004 for W173G, *P* = 0.001 for V198M, all vs. WT. The two-tailed Student's *t*-test was used to determine statistical significance.

Most *XIAP* mutations identified in XLP2 patients are nonsense mutations, frameshift mutations or deletions that cause severe aberrations in the encoded protein or loss of expression (Filipovich et al, 2010; Marsh et al, 2010; Pachlopnik Schmid et al, 2011; Yang et al, 2012). These mutations are positioned throughout *XIAP* and almost invariably interfere with the integrity of the C-terminal RING domain (Fig 1A). Several missense mutations have also been identified in *XIAP*, locating either to the RING or to the BIR2 domain. We report here three novel missense mutations identified in XLP2 patients, c.497G > T (p.R166I), c.620T > C (p.L207P) and c.592G > A (p.V198M), all locating to the BIR2 domain of XIAP [Fig 1A; during preparation of this manuscript the R166I mutations has also been reported by others (Marsh et al, 2013)]. The BIR2-mutated patients presented with EBV-induced HLH including pronounced splenomegaly (XLP phenotype) at age 9 years (p.R166I), 17 years (p.L207P) and 11 years (p.V198M), respectively. Clinical data from one patient (p.R166I) was recently published (P7 in Marsh et al, 2013). He died from acute Graft versus Host Disease (GvHD) and multi-organ failure after peripheral blood stem cell (PBSC) transplantation from an unrelated donor at 14 years. The other patients (p.L207P and p.V198M) had acute EBV-induced HLH and are currently alive and well after the initial treatment with immunosuppressants and rituximab. Clinical details of these patients will be described elsewhere (Carsten Speckmann et al, in preparation). Thus, of nine reported missense mutations, six cause single-amino acid substitutions in the BIR2 domain. Cross-species alignment of the amino acid sequence of the XIAP BIR2 domain and other type-II BIR domains (Eckelman et al, 2008) show that all of the XLP2-mutated residues are highly conserved between IAP proteins and through evolution (Fig 1B). This suggests that the mutated residues are important for the function of the BIR2 domain. Consistently, none of the BIR2 mutations have been reported as single-nucleotide polymorphisms [SNPs; source: Database of SNPs at the National Centre of Biotechnology Information (NCBI; http://www.ncbi.nlm.nih.gov/snp)].

XLP2-causing mutations that affect the RING domain (collectively referred to as XIAP^XLP2-RING^) abrogate XIAP's Ub ligase activity and cause impaired NOD2-dependent immune signalling (Damgaard et al, 2012). This prompted us to investigate if the XLP2-causing BIR2 mutations (collectively referred to as XIAP^XLP2-BIR2^) also affect NOD2 signalling (Fig 1C). XIAP-deficient HCT-116 cells were reconstituted with XIAP^WT^ or XIAP^XLP2-BIR2^ variants and were stimulated with the NOD2 ligand L18-MDP (a lipidated form of MDP with increased potency). As expected, expression of XIAP^WT^ in the XIAP-deficient cells fully restored L18-MDP-induced activation of an NF-κB reporter to the level measured in wild type cells (Fig 1D). Remarkably, none of the six XIAP^XLP2-BIR2^ variants were able to restore activation of the reporter in response to NOD2 activation although expressed at levels comparable to XIAP^WT^ (Fig 1D). XIAP^XLP2-BIR2^ variants were also unable to facilitate NF-κB activation induced by ectopic expression of NOD1 or NOD2 (Fig 1E). This demonstrates that BIR2 mutations, like mutations that affect the XIAP RING domain, severely impair NOD1/2-induced NF-κB activation.

**Figure 2:**
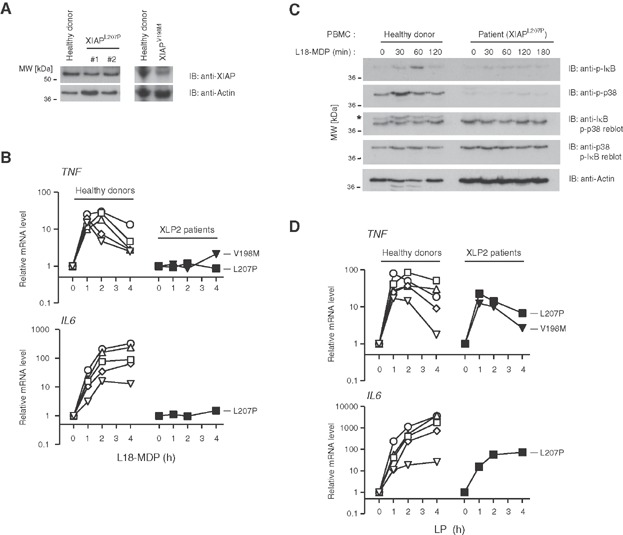
**NOD2 signalling is defective in cells from two XLP2 patients with BIR2 mutations**XIAP levels in XLP2 patient cells. Whole cell lysates of PBMCs from healthy donor and two patients with BIR2 mutations were examined for XIAP levels by immunoblotting. Two separate vials of patient cells were examined and are indicated with 1 and 2.Transcriptional response to NOD2 (B) and TLR4 (D) stimulation in PBMCs. Relative levels of *TNF* and *IL6* mRNA in PBMCs stimulated with L18-MDP (200 ng/mL) or LPS (10 ng/ml) as indicated. Transcription of *TNF* and *IL6* after L18-MDP was impaired in the patient PBMCs compared to cells from healthy donors. Data represent means of one to six experiments in healthy donor cells and two (V198M) or four to six (L207P) experiments in the XLP2 patient cells. *IL6* mRNA was not reliably amplified in cells isolated from the V198M patient. All experiments were performed in duplicate.NOD2 signalling in PBMCs from patient and healthy donor. Cells were examined by immunoblotting for phosphorylation of IκBα and p38 in response to stimulation with L18-MDP (200 ng/mL) as indicated. Asterisk (*) denotes p-p38 signal detectable after re-blotting with anti-IκBα.

To further establish that XLP2-BIR2 mutations impair NOD2-dependent signalling, we obtained peripheral blood mononuclear cells (PBMCs) isolated from two patients with either an XIAP^L207P^ or an XIAP^V198M^ mutation. The expression level of XIAP in PBMCs isolated from the L207P patient was comparable to that of a healthy donor, whereas XIAP levels appeared to be reduced in cells from the V198M patient (Fig 2A). L18-MDP failed to induce transcription of *TNF* and *IL6* in PBMCs from the XLP2 patients (*IL6* was not reliably detectable in the V198M patient cells), whereas transcription was readily induced in cells isolated from five different healthy donors (Fig 2B). Consistently, phosphorylation of IκBα and p38 MAP kinase after NOD2 stimulation was increased only in PBMCs from a healthy donor and not the XIAP^L207P^ patient cells (Fig 2C). This was not due to a general signalling defect in the patient PBMCs because stimulation of Toll-like receptor 4 (TLR4) with lipopolysaccharide (LPS) induced transcription of *TNF* and *IL6* in the patient cells although induction might be slightly reduced compared with healthy donor cells (Fig 2D).

### XLP2-derived XIAP variants partially retain the anti-apoptotic activity

**Figure 3:**
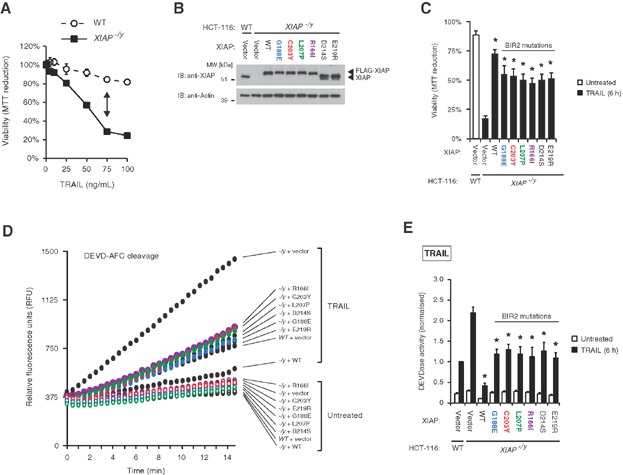
**XIAP BIR2 mutations have minor effect on TRAIL-induced cell death**Viability of WT and XIAP-deficient HCT-116 cells after TRAIL treatment. Cells were treated with indicated concentrations of TRAIL for 24 h and viability was determined by the MTT assay. Data are shown as percentage of vehicle-treated cells and represent mean ± s.e.m. (*n* = 3). Double arrow indicates the difference in sensitivity between WT and XIAP-deficient cells at the concentration used in the following experiments.Expression of XIAP variants in XIAP-deficient HCT-116 cells. Cell lysates were analysed by immunoblotting.XIAP variants protect against TRAIL-induced cell death. Viability of WT and XIAP-deficient HCT-116 cells transfected as indicated and treated with TRAIL (75 ng/mL) for 24 h was determined by the MTT assay. Data are shown as percentage of vehicle-treated cells and represent mean + s.e.m. (*n* = 4–6). (*) Indicates *P* = 2.2E-07 for WT, *P* = 0.0007 for G188E, *P* = 0.0004 for C203Y, *P* = 0.0003 for L207P, *P* = 0.0002 for R166I, *P* = 0.0002 for D214S, *P* = 0.0001 for E219R, all vs. vector in XIAP-deficient cells.Measurement of TRAIL-induced caspase activity. The cleavage of the fluorogenic caspase-3/-7 substrate (DEVD-AFC) was measured in total cell lysates from cells transfected as in (B) and treated with TRAIL (75 ng/mL) for 6 h. Values were corrected for the MTT reduction activity in parallel cultures of untreated cells. (D) Shows linear increase in AFC fluorescence throughout the assay. (E) Shows linear regression of the slope of measurements in (D). Data represent mean + s.e.m. (*n* = 4–6). (*) Indicates *P* = 5.0E-07 for WT, *P* = 0.0004 for G188E, *P* = 0.001 for C203Y, *P* = 0.001 for L207P, *P* = 0.003 for R166I, *P* = 0.006 for D214S, *P* = 0.0008 for E219R, all vs. vector. The two-tailed Student's *t*-test was used to determine statistical significance.

Peripheral T cells from XLP2 patients are often sensitized to activation-induced cell death *in vitro* compared to healthy donor cells (Filipovich et al, 2010; Pachlopnik Schmid et al, 2011; Yang et al, 2012). In line with this, we observed that T cell cultures from the XIAP^L207P^ patient displayed increased rates of apoptosis after treatment with anti-CD3 to cells from a healthy donor (Carsten Speckmann et al, in preparation). The BIR2 domain and the immediate upstream linker contribute to XIAP's anti-apoptotic potential by binding to, and inhibiting, active caspase-3 and caspase-7 (Eckelman et al, 2006; Scott et al, 2005). To investigate if XLP2-BIR2 mutations impact directly on XIAP's ability to inhibit caspases and to protect against apoptosis, we evaluated the ability of XIAP variants to protect *XIAP*^*−/y*^ HCT-116 cells against apoptosis induced by TNF-related apoptosis-inducing ligand (TRAIL). The XIAP-deficient cells were highly sensitive to treatment with TRAIL when compared to wild type HCT-116 cells as previously reported (Cummins et al, 2004), but were rescued by expression of XIAP^WT^ (Fig 3A–C). Expression of XIAP^XLP2-BIR2^ or the IBM-binding pocket mutants XIAP^D214S^ and XIAP^E219R^ protected the cells comparably from TRAIL-induced apoptosis, but slightly less than XIAP^WT^ (Fig 3B and C). Accordingly, the BIR2-mutated XIAP variants reduced caspase-3/-7 activity to a similar level as induced in wild type cells whereas ectopic expression of XIAP^WT^ almost completely blocked TRAIL-induced caspase-3/-7 activity (Fig 3D and E). Together, this suggests that XLP-BIR2 mutations cause severe impairment of NOD1/2-mediated immune signalling, whereas the mutations have less severe consequences for XIAP's anti-apoptotic potential.

### XLP2-BIR2 mutations interfere with XIAP function at the NOD2 signalling complex

**Figure 4:**
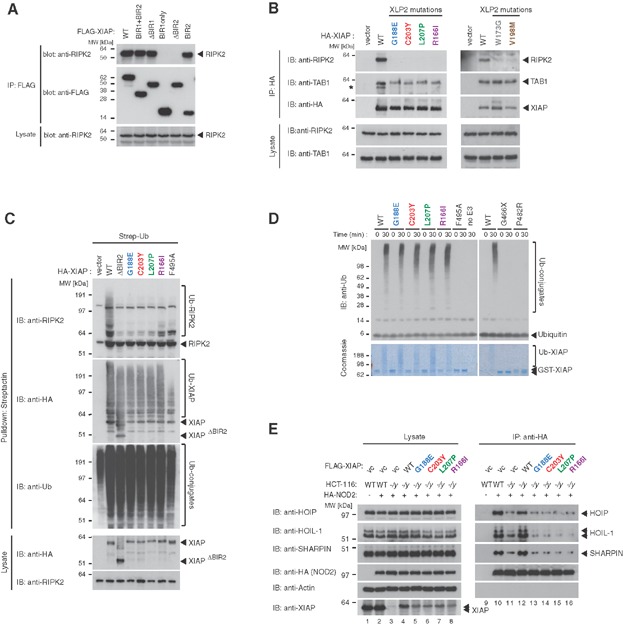
**XLP2-BIR2 mutations interfere with XIAP function at the NOD2 signalling complex**Analysis of RIPK2 binding by XIAP variants and isolated domains. Tagged XIAP was immunoprecipitated from lysates of HEK293T cells transfected with plasmids encoding the indicated FLAG-XIAP fragments (A) or HA-XIAP variants (B). Immunoprecipitates were examined for co-purification of endogenous RIPK2 by immunoblotting. Asterisk by the TAB1 blot indicates non-specific band, possibly cross-reactivity with RIPK2, in the IP.Analysis of XIAP-mediated ubiquitylation of RIPK2. Ub conjugates were purified with StrepTactin-Agarose resin from lysates of HEK293T cells transfected as indicated. Purified material was examined by immunoblotting.Ubiquitin ligase activity of XIAP variants. Purified recombinant XIAP variants were analysed for their Ub ligase activity *in vitro*. Formation of Ub-conjugates was detected by immunoblotting and coomassie staining. Note, that the signal corresponding to GST-XIAP (WT, G188E, C203Y, L207P and R166I) in the coomassie stained gel is lost at 30 min, indicating extensive self-ubiquitylation.Recruitment of LUBAC subunits to NOD2 by XIAP variants. HA-NOD2 was immunoprecipitated from lysates of WT or XIAP-deficient HCT-116 cells expressing HA-NOD2 and FLAG-XIAP (WT or XLP2-BIR2 variants). Immunoprecipitates were examined for co-purification of LUBAC subunits.

Next, we investigated how XLP2-BIR2 mutations affect XIAP function. XIAP is recruited to the NOD2 signalling complex via RIPK2 (Damgaard et al, 2012), suggestively by an interaction between the BIR2 domain in XIAP and RIPK2's kinase domain (Krieg et al, 2009). Accordingly, the interaction between XIAP and RIPK2 required the XIAP BIR2 domain and did not involve other domains of XIAP [Fig 4A; (Krieg et al, 2009)]. Remarkably, all six XLP2-BIR2 mutations abrogated the co-purification of endogenous RIPK2 with XIAP, whereas the binding to the TAK1 adaptor protein TAB1, which binds to the XIAP BIR1 domain, was unaffected by the mutations [Fig 4B; (Lu et al, 2007)].

We reasoned that if XIAP is unable to bind RIPK2 then it is unlikely that XIAP can facilitate the ubiquitylation of RIPK2. Indeed, all tested XLP2-derived BIR2 mutations strongly impaired XIAP-mediated ubiquitylation of endogenous RIPK2 similar to XIAP with a substitution of phenylalanine 495 to alanine (F495A), a mutation previously shown to specifically abrogate XIAP's Ub ligase activity [Fig 4C; (Gyrd-Hansen et al, 2008)]. In contrast to the F495A mutation, the BIR2 mutations did not interfere with XIAP auto-ubiquitylation suggesting that the XIAP^XLP2-BIR2^ variants retain normal Ub ligase activity (Fig 4C). Accordingly, recombinant XIAP^XLP2-BIR2^ variants conjugated Ub chains *in vitro* similar to XIAP^WT^ whereas XIAP^F495A^ failed to detectably conjugate Ub chains (Fig 4D). Two XLP2-derived RING mutations (G466X and P482R) were also examined and, in line with our previous report (Damgaard et al, 2012), both mutations abrogate XIAP's Ub ligase activity and impaired its ability to ubiquitylate RIPK2 [Fig 4D; (Damgaard et al, 2012)]. Thus, although XLP2-derived BIR2 mutations affected RIPK2 ubiquitylation similar to XLP2-derived RING mutations, they do not affect XIAP's intrinsic Ub ligase activity.

An important function of the Ub chains conjugated by XIAP at the NOD2 signalling complex is to enhance the association of the LUBAC subunits HOIP, HOIL-1 and SHARPIN with the complex (Damgaard et al, 2012). To address if LUBAC recruitment to NOD2 is affected by XLP2-BIR2 mutations, we ectopically expressed HA-NOD2 in wild-type cells and in XIAP-deficient cells reconstituted with XIAP^WT^ or XIAP^XLP2-BIR2^ variants. Immunoprecipitation of HA-NOD2 showed that XIAP^XLP2-BIR2^ variants failed to mediate recruitment of LUBAC subunits to the NOD2 complex, whereas XIAP^WT^ mediated the recruitment of LUBAC subunits to similar levels as observed in wild type cells (Fig 4E, compare lanes 10–12, and lane 11 with lanes 13–16). Thus, XLP2-BIR2 mutations specifically abolish RIPK2 binding leading to impaired RIPK2 ubiquitylation and recruitment of LUBAC to the NOD2 signalling complex.

### RIPK2 binding is mediated by residues in XIAP's BIR2 IBM-binding pocket and can be antagonized by Smac

**Figure 5:**
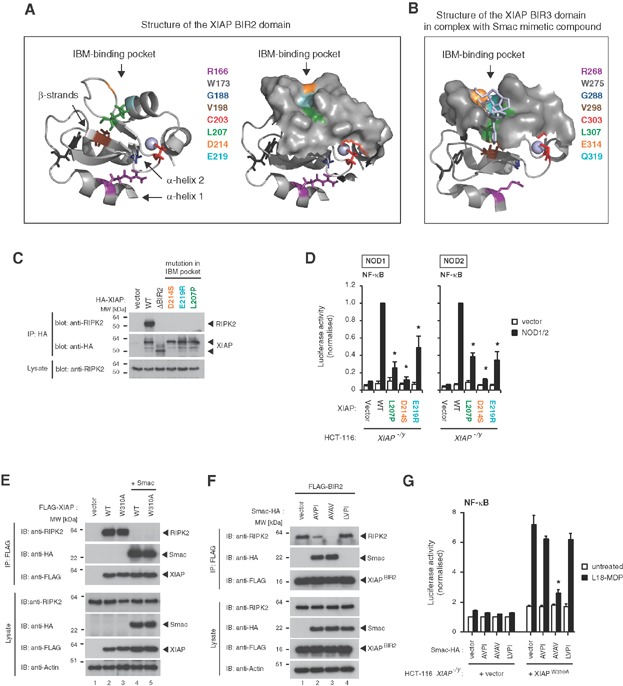
**The BIR2 IBM-binding pocket mediates RIPK2 binding**Cartoon representation of the NMR structure of the BIR2 domain of XIAP. XLP2-BIR2 mutated residues and selected residues of the IBM-binding pocket are in colour and represented with side chains. Zn^2+^-ion is shown as sphere in light blue. The IBM-binding pocket is shown with surface in grey with the residues in the IBM-binding pocket in colour (right panel). PDB id: 1C9Q (Sun et al, 1999).Cartoon representation of the NMR structure of the BIR3 domain of XIAP in complex with a Smac mimetic compound (SMC). The IBM-binding pocket is shown with surface in grey and residues corresponding to the XLP2-BIR2 mutated residues and selected residues of the IBM-binding pocket are shown in colour. Note the SMC (light blue) occupies the cleft of the IBM-binding pocket comprising residues corresponding to L207, D214 and E219 in the BIR2 structure. PDB id: 3EYL (Cossu et al, 2009).Analysis of RIPK2 binding by XIAP variants. HA-XIAP was immunoprecipitated from lysates of HEK293T cells transfected as indicated. Immunoprecipitates were examined for co-purification of endogenous RIPK2 by immunoblotting.NF-κB activity in lysates of XIAP-deficient cells co-transfected with XIAP variants and NOD1 or NOD2 plasmids. Data represent mean + s.e.m. (*n* = 3–5). In NOD1 transfections (*) indicates *P* = 0.0001 for L207P, *P* = 1.3E-06 for D214S, *P* = 0.01 for E219R, all vs. WT. In NOD2 transfections (*) indicates *P* = 0.0001 for L207P, *P* = 3.4E-09 for D214S, *P* = 0.002 for E219R, all vs. WT.Analysis of Smac's effect on the XIAP-RIPK2 interaction. FLAG-tagged XIAP was immunoprecipitated from lysates of HEK293T cells co-transfected as indicated. Immunoprecipitates were examined for co-purification of endogenous RIPK2 by immunoblotting.Analysis of the effect of Smac on NOD2 signalling. NF-κB activity in lysates of XIAP-deficient HCT-116 cells transfected as indicated and stimulated with L18-MDP (200 ng/mL) for 24 h. Data represent mean + s.e.m. (n = 4). Asterisk (*) indicates *P* = 0.0005 for Smac^AVAV^ vs. vector in cells co-transfected with XIAP^W310A^. The two-tailed Student's *t*-test was used to determine statistical significance.

Prompted by these findings, we investigated how the individual XLP2 mutations impact on BIR2 structure and/or function. The NMR structure of the BIR2 domain (Sun et al, 1999) indicates that five of the six XLP2-derived mutations are likely to disturb the overall structure/folding of the BIR2 domain (Fig 5A, left panel): residue C203 is one of four conserved residues that coordinate the Zn^2+^ ion required for folding of the domain (Fig 1B and 5A, left panel; open circles above the BIR2 sequence in Fig 1B denote the Zn^2+^-coordinating residues). W173 and V198 are positioned in the core of the domain and contribute to stabilization of the domain through hydrophobic interactions. G188 is positioned at a conserved tight loop linking α-helix 2 with the three-stranded antiparallel β-sheet that forms the central part of the domain. R166 is located in α-helix 1 with its side chain facing towards α-helix 2 in the core of the domain and is likely to also contribute to the structural stability of the domain. In contrast, L207 is surface-exposed and together with residues D214 and E219 form the cleft of the IBM-binding pocket required for IBM-type interactions [Fig 5A and B, compare the XIAP BIR2 structure with the structure of the XIAP BIR3-Smac mimetic complex; (Cossu et al, 2009; Sun et al, 1999)]. A recent report showed that the E219R mutation impairs RIPK2 binding, whereas mutation of H223 on the ridge of the IBM-binding pocket to a valine increases RIPK2 binding (Krieg et al, 2009). Therefore, we hypothesized that the IBM-binding pocket of the BIR2 domain overlaps with the RIPK2 binding region. To this end, immunoprecipitation of the XIAP^D214S^ and XIAP^E219R^ mutants, similar to XIAP^L207P^, failed to co-purify RIPK2 from HEK293T cell lysates and only poorly restored NOD1- and NOD2-induced NF-κB activation in XIAP-deficient cells (Fig 5C and D). The mature form of Smac harbours an N-terminal IBM (NH_2_-AVPI) enabling it to bind the IBM-binding pockets of XIAP's BIR2 and BIR3 domains (Eckelman et al, 2008; Riedl et al, 2001; Wu et al, 2000). To test if Smac could compete for RIPK2 binding, we co-expressed XIAP and a Ub-Smac fusion protein, which is rapidly cleaved to generate the mature form of Smac (Hunter et al, 2003). In accordance with a previous report (Krieg et al, 2009), Smac efficiently antagonized the interaction between RIPK2 and XIAP^WT^ (Fig 5E, compare lanes 2 and 4). The IBM in Smac (NH_2_-AVPI) is optimal for XIAP BIR3 binding and binds the BIR2 with a lower affinity (Eckelman et al, 2008; Liu et al, 2000; Sweeney et al, 2006). Nonetheless, mutation of the BIR3 IBM-binding-pocket (XIAP^W310A^) did not affect the ability of Smac to compete with RIPK2 for binding to XIAP (Fig 5E, compare lanes 3 and 5). This indicates that Smac can compete with RIPK2 binding through binding to BIR2. Consistently, Smac partially impaired the binding of RIPK2 to the isolated XIAP BIR2 domain (Fig 5F, compare lanes 1 and 2). Screening of combinatorial peptide libraries indicate that the A(V,I)AV sequence is the optimal IBM for the XIAP BIR2 domain (Eckelman et al, 2008; Sweeney et al, 2006). We therefore explored if changing the IBM in Smac from AVPI to AVAV would increase the potency of Smac to compete with RIPK2 for XIAP BIR2 binding. Indeed, co-expression of the BIR2-optimized Smac^AVAV^ antagonized RIPK2 binding more efficiently than did Smac containing the normal AVPI N-terminus, whereas Smac lacking the essential N-terminal alanine (Smac^LVPI^) did not bind the BIR2 and was unable to antagonize RIPK2 binding (Fig 5F). Reflecting the increased potency of Smac^AVAV^ in antagonizing the RIPK2 binding, expression of Smac^AVAV^ impaired the ability of XIAP^W310A^ to restore NOD2-dependent activation of NF-κB in XIAP-deficient cells whereas Smac^AVPI^ or Smac^LVPI^ did not affect NF-κB activation under these conditions (Fig 5G). We conclude that the BIR2 IBM-binding pocket of XIAP overlaps with the RIPK2 binding site.

### Smac mimetic compounds antagonize XIAP function to inhibit NOD1/2-dependent immune signaling

**Figure 6:**
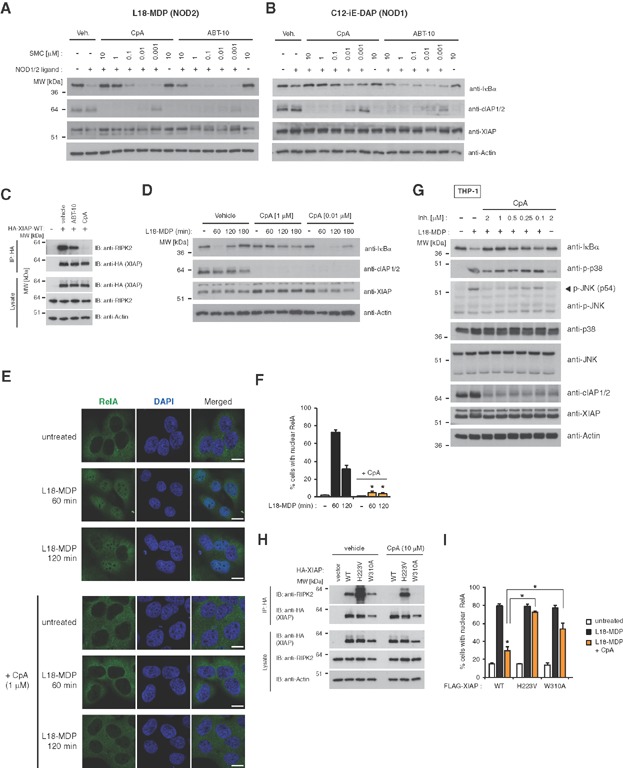
**SMCs inhibit NOD1 and NOD2 signalling in an XIAP-dependent manner**Analysis of NOD1 and NOD2 signalling in the presence of SMCs. U2OS/NOD2 (A and D) or U2OS/NOD1 (B) cells were incubated with the indicated concentrations of SMC for 30 min before treated with L18-MDP or C12-iE-DAP or for 60 min, or as indicated (D). Cell lysates were examined for degradation of IκBα and cIAP1/2 by immunoblotting.Analysis of RIPK2-XIAP binding in the presence of SMCs. Lysates of HEK293T cells transfected with a plasmid encoding HA-XIAP variants as indicated were incubated with 10 μM of the indicated SMCs for 30 min prior to immunoprecipitation of HA-XIAP. Co-purification of endogenous RIPK2 was determined by immunoblotting.Nuclear translocation of NF-κB after NOD2 stimulation. (E) Immunofluorescence analysis of nuclear translocation of the NF-κB subunit RelA in response to L18-MDP stimulation in U2OS/NOD2 cells pre-incubated with CpA for 30 min or not. Scalebar, 10 μm. (F) Quantification of cells with nuclear translocation of NF-κB after L18-MDP stimulation. Data represent mean + s.e.m (n = 3). Each experiment represents >100 cells analysed per experimental condition. Asterisk (*) indicates *P* = 3.2E-05 (60 min) and *P* = 0.003 (120 min) for CpA vs. vehicle treated cells.NOD2 signalling in THP-1 cells treated with CpA. Cells were treated with CpA as indicated and stimulated as in (A). Cell lysates were examined for degradation of IκBα and cIAP1/2, and for phosphorylation of MAP kinases p38 and JNK by immunoblotting.Nuclear translocation of NF-κB after NOD2 stimulation. Quantification of cells with nuclear translocation of NF-κB in cells transfected with plasmids encoding FLAG-tagged XIAP^WT^, XIAP^H223V^ or XIAP^W310A^ before being stimulated as in (E). Data represent mean + s.e.m (*n* = 3–5). Each experiment represents counting of >100 per experimental condition. Asterisk (*) indicates *P* = 1.4E-06 for WT, L18-MDP + CpA vs. L18-MDP, *P* = 9.5E-05 for H223V vs. WT, *P* = 0.01 for W310A vs. WT. The two-tailed Student's *t*-test was used to determine statistical significance.

Prompted by these results, we explored if SMCs could antagonize XIAP function and would interfere with NOD1/2 signalling. SMCs bind with high affinity to type-III BIR domains (BIR3 in cIAP1/2 and XIAP), which causes rapid degradation of cIAP1/2 without affecting XIAP stability (Varfolomeev et al, 2007; Vince et al, 2007). Because cIAP1/2 have been implicated in NOD1/2 signalling (Bertrand et al, 2009), we initially performed titration experiments and correlated cIAP1/2 degradation to the effect of the SMC on signalling. For this, we employed U2OS/FlpIn/TRex cells engineered to respond to L18-MDP or C12-iE-DAP (NOD1 ligand) through single-copy genomic insertion of cDNA encoding HA-tagged NOD1 or NOD2 (here forth termed U2OS/NOD1 and U2OS/NOD2 cells, respectively). The cells were pre-treated with the monovalent ABT-10 or the bivalent Compound A (CpA) SMCs for 30 min before stimulated with NOD1 or NOD2 ligands for 60 min. ABT-10 and CpA both caused degradation of cIAP1/2 when used at 10 nM and above (Fig 6A and B). Despite this, ABT-10 only detectably inhibited degradation of IκBα at a concentration of 10 μM (Fig 6A and B). CpA was significantly more potent but still only inhibited degradation of IκBα after NOD1 and NOD2 stimulation at concentrations 10–100 fold higher than needed to degrade cIAP1/2 (Fig 6A and B). In line with their ability to inhibit NOD2 signalling, CpA more potently displaced RIPK2 from XIAP than did ABT-10 *in vitro* (Fig 6C). Time course analysis showed that CpA at 1 μM impaired L18-MDP-induced degradation of IκBα for at least 3 h whereas CpA at 10 nM only had limited effect on IκBα levels although cIAP1/2 were efficiently depleted (Fig 6D). Accordingly, pre-treatment of U2OS/NOD2 cells with 1 μM CpA almost completely prevented the nuclear translocation of the NF-κB subunit RelA (also termed p65) in response to L18-MDP (Fig 6E and F). We observed similar effects of CpA in THP-1 monocytic cells where pre-treatment with 0.1–2.0 μM CpA impaired degradation of IκBα and phosphorylation of p38 and JNK in a concentration-dependent manner although cIAP1/2 were degraded at all used CpA concentrations (Fig 6G). Together, these data demonstrate that certain SMCs can inhibit NOD1 and NOD2 signalling. Furthermore, they suggested that CpA and ABT-10 inhibit signalling predominantly through antagonizing XIAP's function in the pathway, not by inducing degradation of cIAP1/2.

To directly test this, we compared the ability of XIAP^WT^ to restore NOD2 signalling in the presence of CpA with that of the BIR2-mutated XIAP^H223V^, which shows increased RIPK2 binding compared with XIAP^WT^ [Fig 6H (Krieg et al, 2009)], and the BIR3-mutated XIAP^W310A^. Remarkably, expression of XIAP^H223V^ rendered cells insensitive to CpA, whereas the nuclear translocation of RelA was readily inhibited in cells with ectopic expression of XIAP^WT^ (Fig 6I). Consistent with this, CpA failed to efficiently displace RIPK2 from XIAP^H223V^ *in vitro* under conditions where CpA readily displaced RIPK2 from XIAP^WT^ (Fig 6H). CpA antagonized the interaction between RIPK2 and XIAP^W310A^ *in vitro* similar to XIAP^WT^ (Fig 6H). However, the ability of CpA to inhibit RelA nuclear translocation after NOD2 stimulation was impaired by ectopic expression of XIAP^W310A^, suggesting that inhibition of XIAP function in NOD2 signalling by CpA might involve both the BIR2 and BIR3 domain (Fig 6I).

**Figure 7:**
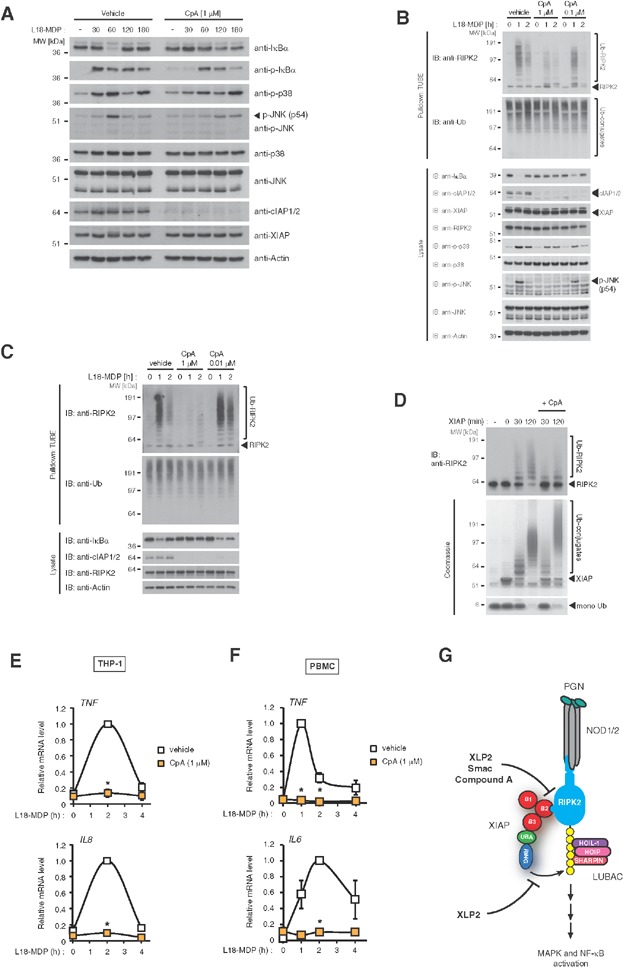
**CpA prevents XIAP-mediated ubiquitylation of RIPK2 and impairs the inflammatory response to NOD2 stimulation**Time course analysis of THP-1 cell lysates incubated with CpA and stimulated with L18-MDP. Cells were incubated with the indicated concentrations of CpA for 30 min before treated with L18-MDP as indicated.Purification of ubiquitin conjugates after NOD2 stimulation. Ubiquitin conjugates were purified from THP-1 cells (B) or U2OS/NOD2 cells (C) incubated with the indicated concentrations of CpA for 30 min before treatment with L18-MDP as indicated. Purified material and cell lysates were examined by immunoblotting.*In vitro* ubiquitylation of RIPK2. Recombinant XIAP was pre-incubated with CpA for 30 min or not before it was added to ubiquitylation buffer containing E1, E2 (UbcH5c), Ub and ATP. The mixture was incubated at 30°C with HA-tagged RIPK2 purified from U2OS cells transfected as indicated. Reactions were stopped by addition of LSB and were analysed by immunoblotting and coomassie staining.Transcriptional response in cells treated with CpA and NOD2 ligand. Relative levels of *TNF* and *IL8* mRNA in THP-1 cells (E) or *TNF* and *IL6* mRNA in PBMCs (F) incubated with CpA for 30 min before stimulation with L18-MDP as indicated. Data represent mean ± s.e.m. (*n* = 3). In THP-1 cells (*) indicates *P* = 0.004 for *TNF*, *P* = 0.0003 for *IL8*, all vs. vehicle. In PBMCs (*) indicates *P* = 0.001 for *TNF* (1 h), *P* = 0.02 for *TNF* (2 h), *P* = 1.2E-06 for *IL8*, all vs. vehicle. The two-tailed Student's *t*-test was used to determine statistical significance.Model of XIAP's role in NOD1/2 signalling. NOD1/2 activation leads to recruitment of XIAP to the signalling complex by RIPK2 through the IBM-binding region in XIAP's BIR2 domain. Mutations in XIAP identified in patients with XLP2 affect either the interaction with RIPK2 or XIAP's RING-dependent Ub ligase activity. In both scenarios, RIPK2 ubiquitylation is abolished, which results in defective signalling and activation of NF-κB. Smac and CpA efficiently abrogate the XIAP-RIPK2 interaction, suggesting that IBM-bearing proteins or IAP antagonistic compounds may modulate NOD1/2-dependent responses. Tandem arrows indicate that additional steps are needed for activation of MAP kinases and NF-κB.

Next, we analysed in detail the functional consequence of CpA treatment on NOD2-dependent signalling processes. Time course analysis of THP-1 cells treated with L18-MDP showed that CpA inhibited IκBα degradation for at least 3 h although some phosphorylation of IκBα was detectable (Fig 7A). CpA also impaired the early peak in activation of MAP kinases p38 and JNK by L18-MDP but was less effective at later time points (Fig 7A). Analysis of RIPK2 ubiquitylation in THP-1 and U2OS/NOD2 cells showed that at 1 µM CpA effectively inhibited L18-MDP-induced ubiquitylation of RIPK2, whereas it had minor effect on RIPK2 ubiquitylation when used at 0.1 µM (THP-1) or 0.01 µM (U2OS/NOD2) (Fig 7B and C). To determine if CpA directly targeted the XIAP-mediated ubiquitylation of RIPK2, we incubated recombinant XIAP with CpA or vehicle and analysed XIAP's ability to ubiquitylate HA-tagged RIPK2 purified from HEK293T cells. Under these conditions, CpA strongly impaired ubiquitylation of RIPK2 by XIAP whereas it did not affect the overall formation of ubiquitin conjugates by XIAP (Fig 7D; note the disappearance of unmodified RIPK2 in the samples where XIAP had not been incubated with CpA). Finally, 1 µM CpA effectively blocked the transcription of NF-κB target genes after NOD2 stimulation in THP-1 cells as well as in healthy donor PBMCs (Fig 7E and F). Together, this demonstrates that pharmacological inhibition of XIAP function by the bivalent SMC Compound A can attenuate NOD1- and NOD2-dependent immune responses.

## DISCUSSION

A direct role for XIAP in innate immune signalling has only recently been described (Bauler et al, 2008; Damgaard et al, 2012; Krieg et al, 2009). However, murine infection models clearly show that XIAP has a critical role in innate immunity and the clinical data on XLP2 patients implicate XIAP function in immune regulation. The clinical presentation of XLP2 varies between individual patients/families, and a clear correlation between disease manifestation and the type of mutation or its position in *XIAP* has not been established. The missense mutations described here were found in patients with varying clinical symptoms ranging from EBV-associated HLH, splenomegaly and hepatitis to severe chronic colitis without evidence of systemic hyperinflammation [(Marsh et al, 2010; Marsh et al, 2013; Worthey et al, 2011), Carsten Speckmann et al, in preparation]. The spectrum of pathologies associated with *XIAP* mutations suggests that defects in XIAP function might contribute to additional disorders characterized by deregulated immune responses such as inflammatory bowel diseases. Polymorphisms in NOD2 that predispose to Crohn's disease confer impaired NF-κB activation in response to NOD2 ligands, which results in chronic inflammation presumably because of an impaired clearance of invading bacteria (Casanova & Abel, 2009). Our data, together with previous studies of XLP2-derived XIAP mutations (Damgaard et al, 2012; Worthey et al, 2011), suggest that impaired NF-κB activation in response to NOD2 (and NOD1) is a common immune defect in patients with XLP2. We therefore speculate that certain *XIAP* mutations or polymorphisms might contribute to susceptibility to Crohn's.

Most XLP2 patients are reported to have little or no expression of XIAP, and XLP2 is occasionally referred to as an “XIAP-deficiency”. However, this is not necessarily the case in patients with missense mutations (Marsh et al, 2010; Pachlopnik Schmid et al, 2011). In fact, the level of XIAP^L207P^ and XIAP^V198M^ in primary PBMCs was comparable to the XIAP level in PBMCs from healthy donors, offering the opportunity to study the functional consequences of individual disease-causing mutations in a particular functional domain of the protein.

XIAP has five functional domains separated by linker sequences; three BIR domains, a UBA domain and a RING domain (Gyrd-Hansen et al, 2008). The fact that all known missense mutations in *XIAP* in XLP2 patients cause amino acid substitution in either the RING domain or in the BIR2 domain points to a critical role of these domains for XIAP's immune regulatory function. Our data demonstrate that six individual BIR2 mutations, similar to RING mutations or XIAP deficiency, impair RIPK2 ubiquitylation by XIAP and strongly attenuate NOD2 signalling. The implication of this is that virtually all described *XIAP* mutations in XLP2 patients, irrespective of whether the mutated protein is expressed or not, will predictably cause impaired RIPK2 ubiquitylation and activation of NF-κB-mediated transcription in response to NOD2 stimulation (and most likely NOD1 stimulation; Fig 7G). Impaired NOD1/2 signalling may thus be a unifying immune defect in these patients and may contribute to the pathogenesis of XLP2, in particular to the inflammatory bowel disease.

We find that RIPK2 binding and NOD2 signalling is antagonized by mutations in the XIAP BIR2 IBM-binding pocket, expression of Smac or incubation with SMCs, CpA in particular. This indicates that the RIPK2 binding region in XIAP BIR2 overlaps with the IBM-binding pocket and might suggest that RIPK2 harbours an IBM-like motif that either is accessible in the unprocessed protein or is exposed after receptor activation. Bioinformatics analysis of the RIPK2 amino acid sequence, however, did not reveal an IBM-like motif. Also, the enhanced binding between XIAP and RIPK2 by the H223V mutation, which is positioned on the ridge of the IBM-binding pocket, argues against a canonical IBM-type interaction because the mutation abrogates the interaction with Smac (Krieg et al, 2009). Nonetheless, our findings show that proteins or chemical compounds that occupy the BIR2 IBM-binding pocket potently attenuate NOD1/2 signalling by antagonising XIAP's binding to RIPK2. This suggests that NOD1/2 signalling might be regulated at the level of XIAP recruitment to the NOD2 signalling complex by IBM-bearing proteins or other factors that interfere with RIPK2 binding. In support of this notion, the inositol phosphatase SHIP-1 was recently reported to negatively regulate NOD1/2 signalling through its interaction with the XIAP BIR2, which inhibited RIPK2 binding (Conde et al, 2012).

Recent reports have highlighted that SMCs may not only be important drugs in anti-cancer treatment, but also could regulate innate immune signalling (Tseng et al, 2010; Vince et al, 2012). Most of the cellular effects of SMCs have been ascribed to the degradation of cIAP1/2, including impairment of the pro-inflammatory response after TLR2 and TLR4 stimulation, NIK-mediated activation of NF-κB activity and the inhibition of IKK activation downstream of TNF-R1 observed with some SMCs (Bertrand et al, 2008; Moulin et al, 2012; Tseng et al, 2010; Varfolomeev et al, 2007; Vince et al, 2007). In these cases SMCs recapitulate the genetic deletion or RNAi-mediated depletion of cIAP1/2. For NOD2-dependent signalling, however, CpA blocked NF-κB activation only at concentrations vastly exceeding those needed for depletion of cIAPs, and our data indicate that CpA functions predominantly through antagonizing XIAP's function in the pathway. This was surprising because cIAP1 and cIAP2 were previously reported to be individually needed for NOD1/2 signalling (Bertrand et al, 2009). Our conclusions, however, are supported by the inability of the monomeric SMC LBW-242 to prevent RIPK2 ubiquitylation after NOD2 stimulation in XIAP-proficient cells (Damgaard et al, 2012) and the poor ability of ABT-10 to inhibit IκBα degradation unless used at very high concentrations. Moreover, mutation of the primary SMC binding site in XIAP (W310A) or introduction of a BIR2 gain-of-function mutation (H223V) rendered NOD2-dependent signalling much less sensitive to inhibition by CpA. In accordance with these observations, a recent study showed that RNAi-mediated knockdown of cIAP1 had limited effect on MDP-induced transcription whereas a chemical IAP antagonist and a Smac N-terminal peptide showed stronger inhibition of transcription (Tigno-Aranjuez et al, 2013). Of note, the IAP antagonist was claimed to be a cIAP1-specific antagonist, which was based on its ability to selectively degrade cIAP1 and does not exclude that it also interfered with XIAP function. We speculate that the inability of SMCs or cIAP1 knockdown to recapitulate the previously reported function of cIAP1/2 in NOD2 signalling, might be explained by the fact that cIAP1-deficient mice carry a passenger mutation inactivating caspase 11 (Kenneth et al, 2012) or possibly by different roles for cIAPs in murine and human cells.

In conclusion, we have uncovered an unexpected but critical role for XIAP's BIR2 domain in immune regulation and demonstrate that XLP2-causing BIR2 mutations have severe consequences for NOD1/2-dependent immune responses. Further, our data suggest that immune processes controlled by XIAP may be regulated by IBM-bearing proteins, and may be attenuated by IAP antagonistic compounds such as certain bivalent SMCs or SMCs targeting the XIAP BIR2 domain.

## MATERIALS AND METHODS

### Patients

The current study was conducted in accordance with the Helsinki Declaration, with informed consent obtained from each patient and the patient's family. Institutional review board approval was obtained prior inclusion of the studied patients (University of Freiburg ethics committee's protocol numbers 143/12). The study is listed in the German Clinical Trial Registry (http://www.drks.de/DRKS00004592).

### Sequence analysis

Multiple alignment analysis was performed with ClustalX. For sequences of the type-II BIR used for analysis, see Supporting Information. Species abbreviations are as follows: Hs (*Homo sapiens*), Mm (*Mus musculus*), Clf (*Canis lupus familiaris*), Bt (*Bos taurus*), Gg (*Gallus gallus*), Xl (*Xenopus laevis*), Xt (*Xenopus tropicalis*), Dr (*Danio rerio*).

### Plasmids and cloning

See Supporting Information.

### Cell lines and human peripheral blood mononuclear cells (PBMCs)

HEK293T and HCT-116 cells were cultured and transfected as previously described (Cummins et al, 2004; Damgaard et al, 2012). U2OS, U2OS/NOD1 and U2OS/NOD2 cells were cultured in DMEM (GIBCO) supplemented with 10% (v/v) FBS (GIBCO) and 0.5% (v/v) Penicillin + Streptomycin (GIBCO) and transfected using FuGENE 6 (Promega, Madison, WI). THP-1 cells were cultured as previously described (Damgaard et al, 2012). EDTA blood samples were taken on site by venopuncture. PBMCs were isolated by Ficoll gradient centrifugation. PBMCs were thawed and cultured in RPMI-1640 (GIBCO) medium supplemented with 10% (v/v) FBS (GIBCO) and 0.5% (v/v) Penicillin + Streptomycin (GIBCO) for 24–72 h before they were subjected to experimental procedures. U2OS/NOD1 and U2OS/NOD2 cell lines were generated as described previously (Fiil et al, 2013). Briefly, human NOD1 and NOD2 cDNAs were cloned into the pcDNA5.1/FRT/TO-2xHA/2xStrep vector (kindly provided by Pascal Meier, ICR, UK). To generate site-specific single-site insertion, pcDNA5.1/FRT/TO-based plasmids were transiently transfected along with pOG44 into U2OS-Flp-In™TREx™ cells (kindly provided by Jakob Nilsson, University of Copenhagen, DK). After hygromycin selection, single cell clones were generated and tested for their ability to activate NF-κB in response to NOD1 and NOD2 ligands. The low levels of NOD1 and NOD2 expression in the absence of doxycycline rendered the cells highly responsive to the cognate NOD1 and NOD2 ligands, respectively, whereas addition of doxycycline resulted in constitutive activation of NF-κB. Throughout the study, the U2OS/NOD1 and U2OS/NOD2 cells were therefore cultured and stimulated in the absence of doxycycline.

### Receptor stimulation and SMC treatment

PBMCs were stimulated for the indicated times with the TLR4 ligand ultrapure LPS from *Escherichia coli* K12 (10 ng/mL; InvivoGen, San Diego, CA) or the NOD2 ligand L18-MDP (200 ng/mL; InvivoGen). Cultured cell lines were stimulated for the indicated times with L18-MDP (200 ng/mL except for immunofluorescence where 1 μg/mL was used), C12-iE-DAP (200 ng/mL; InvivoGen) or TNF (1 ng/mL; R&D systems), which was added directly to the culture medium. SMCs Compound A (CpA) and ABT-10 (kindly provided by TetraLogic Pharmaceuticals) were added to the culture medium 30 min before receptor stimulation.

### Antibodies and affinity resin

All antibodies and affinity reagents were used according to the manufacturers' instructions. For details see Supporting Information.

### Purification of recombinant proteins

GST- and 6xHis-tagged XIAP proteins were expressed in bacteria and purified as previously described (Gileadi et al, 2008; Gyrd-Hansen et al, 2008).

### *In vitro* ubiquitylation assay

2 mM ATP, 80 ng human E1, 1 µg UbcH5c (E2), 3 µg Ub and 0.5 µg GST-tagged XIAP or 5 µg 6xHis-XIAP were mixed in a total volume of 30–50 µL in ubiquitylation buffer (40 mM Tris-HCl pH [7.5], 10 mM MgCl_2_, 0.6 mM DTT) on ice. Reactions were performed at 30°C for 30–120 min and stopped by boiling in LSB. Samples were subjected to SDS-PAGE and proteins were visualized by Coomassie blue staining or immunoblotting. For characterization of SMCs, 6xHis-tagged XIAP was incubated with CpA on ice for 30 min at a 1:2 molar ratio before added to the ubiquitylation buffer. The reaction mixture was subsequently incubated with anti-HA-agarose-coupled HA-tagged RIPK2 isolated from transiently transfected U2OS cells. Reactions were stopped and analysed as described above.

### Caspase-3/-7 protease activity assay

HCT-116 cells were cultured in 96-well plates and transfected as indicated 48 h before treatment with TRAIL for 6 h. Cells were lysed in 55 µL lysis buffer (0.5% Triton X-100, 10 mM Tris-HCl pH 7.5, 8 mM DTT and 1 mM pefabloc) for 30 min on ice. 50 µL lysate was transferred to a black 96-well plate together with an equal volume of 2× caspase reaction buffer (50 µM Ac-Asp-Glu-Val-Asp-(7-amino-4-trifluoromethylcoumarin) (DEVD-AFC; Enzo Life Sciences, Farmingdale, NY), 100 mM HEPES pH 7.5, 20% glycerol, 0.5 mM EDTA, 0.1% CHAPS, 5 mM DTT and 1 mM pefabloc). The fluorescence intensity of liberated AFC was measured as relative fluorescence units (RFU) every 30 s for 15 min (excitation, 400 nm; emission 489 nm) on a FLUOstar Omega Microplate Reader (BMG LABTECH). DEVDase activity was calculated as the slope of the increase in RFUs over 15 min.

### MTT reduction assay

HCT-116 cells were cultured in 96-well plates and transfected as indicated 48 h before treatment with TRAIL for 24 h. The medium was aspirated and 100 µL fresh medium was added together with 25 µL 3-(4,5-dimethylthiazol-2-yl)-2,5-diphenyltetrazolium bromide (MTT; 5 mg/mL dissolved in PBS) and the cells were left to incubate for 1.5 h at 37°C. Afterwards, 100 µL solubilization buffer (20% SDS (w/v) dissolved in 50% *N*,*N*-dimethylformamide) was added and samples were left to incubate over night. Absorbance at 590 nm was read with a reference filter of 620 nm. Individual experiments were performed in duplicate.

### Immunoprecipitation and pulldown

HEK293T, U2OS or HCT116 cells were transfected and treated as indicated. Cells were lysed in IP buffer (25 mM HEPES pH 7.4, 150 mM KCl, 2 mM MgCl_2_, 1 mM EGTA, 0.5% (v/v) Triton X-100) supplemented with 2-Iodoacetamide (final concentration 50 mM) and PhosSTOP (Roche Diagnostics) for 30 min on ice. Lysates were cleared by centrifugation and were incubated at 4°C for 2–18 h with antibody-coupled beads. Beads were washed four times in 500 μL ice-cold IP buffer and bound material eluted with either 0.2 M glycine, pH 2.5 (HA IP and FLAG IP) or 1× LSB (Strep pulldown). For purification of endogenous Ub-conjugates, Tandem Ubiquitin Binding Entities (TUBE) were used as previously described (Damgaard et al, 2012).

### Luciferase reporter assays

Cells were co-transfected with the NF-κB luciferase reporter construct pBIIX-luc and a thymidine kinase-renilla luciferase (TK-renilla-Luc) construct for normalization of transfection efficiency. Cells were either co-transfected with additional plasmids or treated with compounds as indicated elsewhere and luciferase assays were performed as previously described (Damgaard et al, 2012). Individual experiments were performed in duplicate.

### Quantitative RT-PCR

Total RNA was isolated from PBMCs using RNeasy Mini Kit (Qiagen) and DNase digestion was performed on-column with the RNase-Free DNase Set (Qiagen) according to manufacturer's protocol. Total RNA was reverse transcribed with SuperScript® III Reverse Transcriptase (Invitrogen) and oligo(d)T primers, in the presence of RNasin® (Promega). QPCR was performed using Brilliant III Ultra-Fast SYBR® Green QPCR Master (Agilent Technologies). All experiments were performed as two technical replicates. For primer sequences, see Supporting Information.

### Immunofluorescence staining and microscopy

Cells were fixed in 4% formaldehyde, permeabilized with PBS containing 0.2% Triton X-100 for 5 min and incubated with primary antibodies diluted in DMEM for 1 h at room temperature. After staining with secondary antibodies (Alexa Fluor 488 goat anti-rabbit IgG (A11008) and Alexa Fluor 568 goat anti-mouse IgG (A11005), Life Technologies) for 30 min, cover slips were mounted in Vectashield mounting medium (Vector Laboratories, Burlingame, CA) containing the DNA stain DAPI. Images were acquired with an LSM 780 confocal microscope (Carl Zeiss Microimaging) mounted on Zeiss Axiovert 100 M equipped with Plan-Apochromat ×40, 1.3 numerical aperture (NA) oil-immersion objective, using standard settings. Image acquisition and analysis was carried out with ZEN2010 software. No image processing was used. For data quantification, at least 100 FLAG (XIAP)-positive cells per experimental condition were counted in each experiment.

### Statistical analysis

The two-tailed Student's *t*-test was used to determine statistical significance. Error bars represent standard error of the mean (s.e.m.).

Mutations in *XIAP*, the gene encoding X-linked Inhibitor of Apoptosis (XIAP), cause X-linked lymphoproliferative syndrome type-2 (XLP2). XLP2 is an immunodeficiency characterized by susceptibility to Epstein-Barr Virus (EBV) infection with a high risk of HLH, severe chronic colitis, hepatitis and/or persistent splenomegaly.

XIAP is a multifunctional protein best known for its ability to prevent apoptosis by directly inhibiting caspase-3 and -7. Recently, XIAP has emerged also as an essential transducer of pro-inflammatory signalling downstream of the cytosolic bacterial sensors NOD1 and NOD2. Here, XIAP via its RING domain facilitates non-degradative ubiquitylation of the adaptor kinase RIPK2 to activate MAP kinases and NF-κB transcription factors that, in turn, orchestrate an inflammatory response.

The molecular functions of XIAP that are compromised in XLP2 patients, and how this might contribute to the pathogenesis of XLP2 remains poorly understood.

RESULTS:

We here uncover that the region in *XIAP* encoding the BIR2 domain of the protein is a hotspot for missense mutations in XLP2 patients. Analysis of the mutations shows that they cause severe impairment of NOD2 signalling and transcription of pro-inflammatory mediators. Molecularly, we find that the BIR2 mutations interfere with XIAP's binding to RIPK2, thereby preventing ubiquitylation of RIPK2. Employing a small-molecule antagonist of IAP proteins (termed Compound A) that interferes with XIAP's binding of RIPK2, we demonstrate that NOD1- and NOD2-signalling dependent innate immune signalling can be modulated pharmacologically.

IMPACT:

Our results indicate that the interaction between the BIR2 domain in XIAP and RIPK2 is a central regulatory point for innate immune signalling in response to NOD1 and NOD2 stimulation. Because virtually all identified XLP2-associated mutations affect either the BIR2 domain or the integrity of the XIAP RING domain, our results point to impaired NOD2-dependent (and presumably also NOD1-dependent) signalling as a unifying immune defect in XLP2 patients, suggesting that this may contribute to some aspects of the disease.

## Author contributions

RBD and BKF performed the majority of experiments, analysed the data, and contributed to writing of the manuscript. CS, UzS and SE identified patients with novel *XIAP* mutations, analysed patient material and helped in writing the manuscript. MY and PJJ analysed patient material and cell lines, and contributed to analysis of data and correction of the manuscript. SBJ and NM contributed to acquisition of micrographs, analysis of data, and correction of the manuscript. MGH designed the study, analysed the data and wrote the manuscript.

## For more information

- Author websites:
- http://www.cpr.ku.dk/groups/diseasebiology/ubiquitin/
- http://www.gyrd.dk
- http://www.uniklinik-freiburg.de/cci/live/index_en.html
- Study information:
- http://www.uniklinik-freiburg.de/cci/live/klinische-studien/xiap_en.html
- Clinical Trial Registry:
- http://www.drks.de/DRKS00004592
- http://apps.who.int/trialsearch/trial.aspx?trialid=DRKS00004592

## Supporting Information

As a service to our authors and readers, this journal provides supporting information supplied by the authors. Such materials are peer reviewed and may be re-organized for online delivery, but are not copy-edited or typeset. Technical support issues arising from supporting information (other than missing files) should be addressed to the authors.
