# Supplementary material for: Disease-causing mutations in the XIAP BIR2 domain impair NOD2-dependent immune signalling
Source: EMBO Mol Med. 2013 Jul 1;5(8):1278–95. doi: 10.1002/emmm.201303090 (PMC3944466; doi:10.1002/emmm.201303090)
Supplement: Supplementary file 1 [file emmm0005-1278-SD1.pdf]

Supporting Information

Disease-causing mutations in the XIAP BIR2 domain impair NOD2-dependent immune signalling

Rune Busk Damgaard, Berthe Katrine Fiil, Carsten Speckmann, Monica Yabal, Udo zur Stadt, Simon Bekker-Jensen, Philipp J. Jost, Stephan Ehl, Niels Mailand, Mads Gyrd-Hansen

TABLE OF CONTENTS

Supporting Materials and methods

- Sequence analysis
- Plasmids and cloning
- Antibodies and affinity reagents
- Quantitative RT-PCR

SUPPORTING MATERIALS AND METHODS

Sequence analysis

BIRC2 Hs(NM_01166), BIRC2 Mm(NM_007465), BIRC2 Clf(NM_001048023), BIRC2 Bt(NM_001035293), BIRC2 Gg(NM_001007822), BIRC2 Xl(NM_001093264), BIRC2 Dr(NM_194395), BIRC3 Hs(NM_001165), BIRC3 Mm(NM_007464), BIRC3 Clf(NM_001080725), BIRC4 Hs(NM_001167), BIRC4 Mm(NM_009688), BIRC4 Clf(DQ225116), BIRC4 Bt(XM_583068), BIRC4 Gg(NM_204588), BIRC4 Dr(NM_194396), XIAP (NM_001095614), BIRC5 Hs(NM_001168), BIRC5 Mm(NM_009689), BIRC5 Clf(NM_001003348), BIRC5 Bt(NM_001001855), BIRC5 Gg(NM_001012318), BIRC5 Xt(NM_001044483), BIRC6 Hs(NM_016252), BIRC6 Mm(NM_007566), BIRC6 BIRC6 Clf(XM_848959), Bt(NM_001206582), BIRC6 Gg(XM_419512), BIRC6 Xt(XM_002934459), BIRC6 Dr(XM_001336866). Species abbreviations are as follows: Hs (*Homo sapiens*), Mm (*Mus musculus*), Clf (*Canis lupus familiaris*), Bt (*Bos taurus*), Gg (*Gallus Gallus*), Xl (*Xenopus laevis*), Xt (*Xenopus tropicalis*), Dr (*Danio rerio*)

Plasmids and cloning

The NF-κB luciferase reporter plasmids, pBIIX-Luc and TK-renilla-Luc, pcDNA3-3xHA-ubiquitin, pEBB-FLAG-XIAP, pcDNA3-3xHA-XIAP, pcDNA-3xHA-XIAP^{F495A}, and pcDNA-3xHA-XIAP^{G488Stop} have been described previously (Damgaard et al, 2012; Gyrd-Hansen et al, 2008). pEF-XIAP^{WT}-FLAG, pEF-XIAP^{BIR1+2}-FLAG, pEF-XIAP$^{\Delta BIR1}$-FLAG, pEF-XIAP$^{BIR1\text{-}only}$-FLAG, pEF-XIAP$^{\Delta BIR2}$-FLAG, pEF-XIAP$^{BIR2\text{-}only}$-FLAG, pEF-XIAP^{D214S}-FLAG, pcDNA5-FRT-TO-XIAP-FLAG, and pcDNA5-FRT-TO-XIAP^{W310A}-FLAG were kind gifts from Dr.

J Silke (Walter and Eliza Hall Institute for Medical Research, Melbourne, Australia) and have been described previously (Silke et al, 2002). pcDNA-3xHA-XIAP$^{\Delta BIR2}$ and pcDNA-3xHA-XIAP^{D214S} were generated using PCR amplification products from the corresponding pEF constructs inserted into pcDNA3-3xHA. Additional XIAP mutations described were generated by PCR-based site-directed mutagenesis. The Ub-Smac construct has been described before (Hunter et al, 2003). Ub-Smac^{AVAV} and Ub-Smac^{LVPI} variants were generated using site-directed mutagenesis. All constructs have been verified by DNA sequencing. Full length NOD2 was amplified by PCR from a cDNA clone and inserted into pcDNA5.

Antibodies and affinity reagents

The following antibodies and affinity reagents were used according to the manufacturers' instructions: rat monoclonal anti-HA (#11867423991; Roche Diagnostics, Burgess Hill, UK), M2 anti-FLAG conjugated to HRP (A8592), M2 anti-FLAG-agarose resin (A2220), anti-HA-agarose resin (A2095) and rabbit polyclonal anti-HOIP (SAB2102031; Sigma-Aldrich, Gillingham, UK), mouse monoclonal anti-XIAP (#610716; BD Biosciences, San Jose, CA), mouse monoclonal anti-XIAP (#28151; Abcam, Cambridge, MA; Fig 3E), mouse monoclonal anti-ubiquitin (IMG-5021; Imgenex, San Diego, CA), rabbit polyclonal anti-RIPK2 (sc-22763; Santa Cruz Biotechnology, Santa Cruz, CA), rabbit polyclonal anti-SHARPIN (#14626-1-AP; ProteinTech, Chicago, IL), monoclonal anti-β-actin (MAB1501; Chemicon, Millipore, Billerica, MA), mouse monoclonal anti-HOIL-1 (Haas et al, 2009), rabbit polyclonal antibodies to TAB1 (#3226), p38 MAP Kinase (#9215) and IκBα (#9242), mouse monoclonal anti-phospho-p38 MAP Kinase (Thr180/Tyr182; #9216), and rabbit polyclonal anti-phospho-IκBα (Ser32; #2859) (Cell Signaling Technology,

Danvers, MA), mouse monoclonal anti-pan-cIAP (MAB3400; R&D Systems, Minneapolis, MN), HRP-conjugated rabbit polyclonal anti-mouse IgG (P026002-2; Dako, Glostrup, Denmark), HRP-conjugated polyclonal goat anti-rabbit IgG (PI-1000; Vector Laboratories, Burlingame, CA), HRP-conjugated goat polyclonal anti-rat IgG (#31470; Pierce, Thermo Scientific, Rockford, IL), Strep-Tactin-Sepharose resin (#2-1201-010; IBA, Gottingen, Germany). For immunofluorescence, rabbit polyclonal anti-NF-κB p65 (sc-372; Santa Cruz Biotechnology) and mouse monoclonal anti-FLAG M2 (F1804; Sigma-Aldrich) antibodies were used.

Quantitative RT-PCR

cDNA was amplified with the following primer pairs:

Hypoxanthine phosphoribosyltransferase (*HPRT*; used as reference for normalisation):

5’-AGCCAGACTTTGTTGGATTTG-3’
5’-TTTACTGGCGATGTCAATAGG-3’,

TNF: 5’-TGCTGCAGGACTTGAGAAGA-3’
5’-GAGGAAGGCCTAAGGTCCAC-3’,

IL6: 5’-AAAGAGGCACTGGCAGAAAA-3’
5’-TTTCACCAGGCAAGTCTCCT-3’,

IL8: 5’-TCTGGCAACCCTAGTCTGCT-3’
5’-AAACCAAGGCACAGTGGAAC -3’.
