# Supplementary material for: Disease-causing mutations in the XIAP BIR2 domain impair NOD2-dependent immune signalling
Source: EMBO Mol Med. 2013 Jul 1;5(8):1278–95. doi: 10.1002/emmm.201303090 (PMC3944466; doi:10.1002/emmm.201303090)
Supplement: Supplementary file 2 [file emmm0005-1278-SD2.pdf]

EMBO
Molecular Medicine

Disease-causing mutations in the XIAP BIR2 domain impair NOD2-dependent immune signalling

Rune Busk Damgaard, Berthe Katrine Fiil, Carsten Speckmann, Monica Yabal, Udo zur Stadt, Simon Bekker-Jensen, Philipp J. Jost, Stephan Ehl, Niels Mailand, Mads Gyrd-Hansen

Corresponding author: Mads Gyrd-Hansen, University of Copenhagen

Review timeline:

Submission date:	28 May 2013
Accepted:	03 June 2013

Editor: Céline Carret

Transaction Report:

Please note that the manuscript was previously reviewed at another journal and the reports were taken into account in the decision making process at EMBO Molecular Medicine. Since the original reviews are not subject to EMBO's transparent review process policy, the reports and author response cannot be published.
